# Cumulative biomedical risk and social cognition in the second year of life: prediction and moderation by responsive parenting

**DOI:** 10.3389/fpsyg.2015.00354

**Published:** 2015-04-01

**Authors:** Mark Wade, Sheri Madigan, Emis Akbari, Jennifer M. Jenkins

**Affiliations:** ^1^Department of Applied Psychology and Human Development, University of TorontoToronto, ON, Canada; ^2^Atkinson Centre for Society and Child Development, Fraser Mustard Institute for Human Development, University of TorontoToronto, ON, Canada

**Keywords:** social cognition, biomedical risk, parenting, maternal responsivity, risk-resilience

## Abstract

At 18 months, children show marked variability in their social-cognitive skill development, and the preponderance of past research has focused on constitutional and contextual factors in explaining this variability. Extending this literature, the current study examined whether cumulative biomedical risk represents another source of variability in social cognition at 18 months. Further, we aimed to determine whether responsive parenting moderated the association between biomedical risk and social cognition. A prospective community birth cohort of 501 families was recruited at the time of the child’s birth. Cumulative biomedical risk was measured as a count of 10 prenatal/birth complications. Families were followed up at 18 months, at which point social-cognitive data was collected on children’s joint attention, empathy, cooperation, and self-recognition using previously validated tasks. Concurrently, responsive maternal behavior was assessed through observational coding of mother–child interactions. After controlling for covariates (e.g., age, gender, child language, socioeconomic variables), both cumulative biomedical risk and maternal responsivity significantly predicted social cognition at 18 months. Above and beyond these main effects, there was also a significant interaction between biomedical risk and maternal responsivity, such that higher biomedical risk was significantly associated with compromised social cognition at 18 months, but only in children who experienced low levels of responsive parenting. For those receiving comparatively high levels of responsive parenting, there was no apparent effect of biomedical risk on social cognition. This study shows that cumulative biomedical risk may be one source of inter-individual variability in social cognition at 18 months. However, positive postnatal experiences, particularly high levels of responsive parenting, may protect children against the deleterious effects of these risks on social cognition.

## Introduction

Social cognition is the set of cognitive processes related to social understanding and behavior. The capacity to understand human actions in terms of the psychological states that motivate behavior is a fundamental component of social cognition. While social cognition is broadly defined and includes a number of cognitive processes, it is generally well accepted that by the second year of life children evince many basic social-cognitive competencies, including an understanding of others’ goals (Csibra et al., 2003), intentions (Behne et al., 2005), desires (Repacholi and Gopnik, 1997), emotions (Moses et al., 2001), and perhaps even beliefs (Buttelmann et al., 2009). The ability to understand others’ mental states manifests itself in a number of overt behaviors in the second year of life, many of which are used to index early social cognition. For instance, by 18 months children engage in regular bouts of joint attention (Tomasello et al., 2005; Tomasello and Carpenter, 2007), empathy (Roth-Hanania et al., 2011), cooperation (Brownell et al., 2006; Warneken et al., 2006; Warneken and Tomasello, 2007), and self-recognition (Nielsen and Dissanayake, 2004; Brownell et al., 2007). These social-cognitive skills rely on the capacity to differentiate self from other (Asendorpf et al., 1996; Lewis, 2003), and it has been suggested that children’s emergent aptitude for understanding intentions may play a critical role in their ability to engage successfully in these behaviors (Moore, 2007; Knoblich and Sebanz, 2008).

Although social cognition develops progressively over childhood (Gergely and Csibra, 2003; San Juan and Astington, 2012; Thoermer et al., 2012), there are important individual differences in early social cognition that have a bearing on later skills such as theory of mind (Legerstee, 2005; Aschersleben et al., 2008; Wellman et al., 2008). This variability in social reasoning can also be observed in adolescence (Moriguchi et al., 2007; Dumontheil et al., 2010). Longitudinal studies show that individual differences in social cognition are quite stable (Pons and Harris, 2005) and are related to multiple developmental outcomes (Frischen et al., 2007; Fiske and Taylor, 2013). For instance, theory of mind ability has been linked to children’s academic achievement (Blair and Razza, 2007), behavioral problems (Hughes and Ensor, 2006), and social competence (Razza and Blair, 2009). Accordingly, it is important to identify sources of variability in early social cognition, which may exert downstream effects on multiple domains of functioning.

To date, the preponderance of literature on predictors of social cognition has focused on contextual factors such as family processes and socioeconomic variables. For instance, Dunn et al. (1991) have shown that mothers’ mental state discourse and family socioeconomic status (SES) at 33 months are associated with emotion understanding at 40 months. The effect of socioeconomic factors on individual differences in theory of mind has been replicated in numerous investigations (Holmes et al., 1996; Shatz et al., 2003). Moreover, the effect of parenting behavior on social cognition is one of the most robust findings in the literature on social cognition (Pears and Moses, 2003; de Rosnay and Hughes, 2006; Ruffman et al., 2006). Also relevant are child-level factors such as gender, with females demonstrating overall better social cognition than males (Dunn et al., 1991). One of the strongest factors associated with social cognition is language ability (Astington and Jenkins, 1999; Cutting and Dunn, 1999; de Rosnay and Harris, 2002; Pons et al., 2003), which may play both a communicational and representational role in social cognition (see Dunn and Brophy, 2005). Thus, there appears to be a range of known environmental and child-specific factors that contribute to individual differences in social cognition across childhood.

Importantly, much of the existing literature has focused on predictors of social cognition in preschool and school-age children. Relatively less is known about the factors associated with social cognition at earlier stages of development. However, recent studies suggest that, as early as the second year of life, there may be multiple influences on social cognition, such as cumulative social disadvantage, maternal sensitivity, and language ability (Wade et al., 2014c) as well as oxytocin genetic variability (Wade et al., 2014b) and pregnancy hypertension (Wade and Jenkins, 2014). These results are consistent with the manifold biopsychosocial correlates of social cognition observed in preschool children. However, across all studies there remains a substantial proportion of unexplained residual variance, suggesting the presence of currently unspecified influences on social cognition. The goal of the current study was to examine whether early biomedical risk, or the occurrence of combined pre- and perinatal complications, represented another source of variability in social cognition in the second year of life. Further, supposing that such a relationship exists, and consistent with the known effects of contextual factors on social-cognitive development, we aimed to determine whether positive postnatal interpersonal experiences with caregivers (i.e., responsive parenting) protected children against these potentially adverse biomedical risks.

Specific biomedical risk factors for early social cognition have been vastly understudied. In one recent study, Wade and Jenkins (2014) demonstrated that pregnancy hypertension is associated with lower social cognition at 18 months, as well as theory of mind ability in the preschool period. Another recent study showed that birth weight was positively associated with theory of mind at age 4.5 in a typically developing sample (Wade et al., 2014a). Together, these studies provide preliminary evidence that pre- and perinatal factors may be involved in a mechanism through which early fetal stress impinges on healthy brain development that supports social cognition. Aside from these findings, however, little is known about the role of biomedical factors on social cognition in the second year of life.

Indirect evidence for a role of early medical complications on social cognition comes from research showing that such factors are related to the risk of neurodevelopmental and psychiatric disorders characterized by deficits in social cognition. For instance, a comprehensive review by Kolevzon et al. (2007) revealed that the most prominent obstetric complications associated with risk for autism spectrum disorder (ASD) included birth weight, gestational age, as well as intrapartum hypoxia. Obstetrical complications have also been linked to the risk for schizophrenia (Geddes and Lawrie, 1995; Verdoux et al., 1997), eating disorders (Cnattingius et al., 1999), early onset affective disorders (Guth et al., 1993), substance abuse (Sydsjö, 2011), attention-deficit hyperactivity disorder (Milberger et al., 1997; Bhutta et al., 2002), and conduct, oppositional, and internalizing problems (Cohen et al., 1989). In a prospective follow-up study, Buka et al. (1993) suggested that fetal hypoxia was the common underlying mechanism and was the strongest predictor of later cognitive and psychiatric difficulties. Several maternal pathologies during pregnancy have been linked to perinatal hypoxia–ischemia, such as infections, diabetes, hypertension, and thyroid problems (Shah, 2001; Kurinczuk et al., 2010; Teramo, 2010; Stanek, 2013). Thus, it is conceivable that these biomedical factors increase the risk of hypoxic-ischemic events which compromise development in key social-cognitive domains that typify neurodevelopmental and psychiatric conditions.

Two important points deserve consideration here. The first is that early biomedical complications likely produce a continuum of postnatal biopsychosocial-health variability, rather than just the extremes of problems (Pasamanick and Knobloch, 1961). This means that we should expect to observe individual differences in discrete social, cognitive, and emotional phenotypes that characterize neurodevelopmental and psychiatric conditions as a function of biomedical risk. Second, the existing research is limited in differentiating between the effect of different types of prenatal/birth complications on developmental outcomes (Allen et al., 1998). Indeed, there are a variety of biomedical complications that can occur during the pre-, peri-, and neonatal period, including those related to maternal physical health (e.g., endocrine/inflammatory diseases), intrapartum events (e.g., physical trauma), perinatal problems (e.g., low birth weight, prematurity), and immediate postpartum factors (e.g., anoxia or hematological problems demanding use of specialized care). However, it may be difficult to ascertain the effect of each individual risk on children’s outcomes, particularly in epidemiological samples where the prevalence of certain conditions may be too low to provide powerful estimates and the measurement is not sufficiently detailed to effectively partition risks. As a result, one approach that may be useful is the *cumulative risk* approach. The overarching idea behind cumulative risk measures is that, rather than a single and specific risk, it is the aggregation of multiple risks that compromises development (Dong et al., 2004; Flouri and Kallis, 2007; Burchinal et al., 2008). Indeed, it has been repeatedly demonstrated that cumulative risk indices are more stable than individual risk measures (Burchinal et al., 2000), and explain more variance in child outcomes than risks examined in isolation (Deater-Deckard et al., 1998; Atzaba-Poria et al., 2004; Flouri and Kallis, 2007; Evans et al., 2013).

While the cumulative risk approach has been applied widely within the psychosocial domain, its application to prenatal/birth risks is far less common. Nonetheless, existing research indicates that the accumulation of biomedical risks in the pre- and perinatal period is detrimental to children’s socioemotional, intellectual, and motor functioning (Laucht et al., 1997), as well as their visual memory (Levy-Shiff et al., 1994) and attentional control (Carmody et al., 2006). However, these studies have generally assessed the effect of medical complications in children born preterm, which represents a group of already at-risk children who may be particularly vulnerable to negative outcomes. The effect of biomedical risk (i.e., prenatal/birth complications) on social cognition in the general community remains unexplored. Further, no study has examined how enriched postnatal experiences may protect against early biomedical risk on social cognition.

Parental inputs are believed to foster social cognition owing to their role in providing children with the linguistic, representational, and reflective material needed to understand others’ minds (Fernyhough, 2008). Further, it has been demonstrated that positive experiences with caregivers exert a *protective* influence on children (Rutter, 1987; Brody et al., 2002; Burchinal et al., 2006). Protective in this regard does not mean avoiding risk, but persevering in the face of it. These ‘moderation’ models are typically examined by determining whether the association between two variables depends on the level of a third variable, with the risk variable (e.g., biological risk) being less predictive of the outcome when the presumed protective factor is present. Surprisingly, there is little existing research on parenting as a protective factor in regard to the development of social-cognitive capacities, or as a moderator of the association between biological risk and children’s outcomes in general. The limited research to date, however, does suggest that certain aspects of parenting may buffer children against early biomedical risk. For example, Laucht et al. (2001) found that responsive parenting moderated the effect of birth weight on school-aged children’s hyperkinetic and internalizing problems, and Voigt et al. (2013) showed that the effect of neonatal distress on children’s negative affectivity at 12 months depended on the level of parenting stress, with lower levels of stress protecting against neonatal problems. Finally, another interesting study examining children’s executive functioning – a neurocognitive skill that is developmentally linked to social cognition – showed that the effect of neurobiological risk (i.e., direct measurement from neonatal medical records, e.g., need for oxygen/ventilation) on executive functioning was most prominent in socioeconomically disadvatanged children (Ford et al., 2011). Thus, to build on this literature, and in line with risk-resiliency models of development (Luthar et al., 2000; Masten et al., 2009; Jenkins et al., in press), the current study aimed to determine whether, given an association between cumulative biomedical risk and social cognition, responsive parenting moderated this association. Specifically, it was hypothesized that higher levels of biomedical risk would be associated with lower social cognition at 18 months; however, if children received high levels of responsive parenting, the effect of biomedical risk on social cognition would be attenuated.

## Materials and Methods

### Participants

Participants came from the intensive sample of the Kids, Families, Places Study (iKFP; http://kfp.oise.utoronto.ca/). All women giving birth in Toronto and Hamilton, Ontario, between April 2006 and September 2007 were considered for participation. Families were recruited through a program called *Healthy Babies Healthy Children*. Parents of all registered newborns were contacted within several days of the child’s birth. Inclusion criteria for the iKFP study included the presence of an English-speaking mother, a newborn >1500 g, at least two children who are <4 years, and families agreeing to be filmed in the home. Of those contacted, 34% of families agreed to take part in the study. Reasons for non-enlistment included refusals and an inability to contact families from public health’s information. The University of Toronto Research Ethics Board approved all procedures for this investigation, including informed consent.

We compared our sample (*N* = 501) with the general population of Toronto and Hamilton using 2006 Census Data, limiting the census to women between 20 and 50 years and having at least one child. Families were compared based upon immigrant status, number of persons in the home, family structure, maternal personal income, and educational level. Based on these comparisons, iKFP was similar to the general population on family size (*M* = 4.52, SD = 1.01 vs. *M* = 4.13, SD = 1.22) and personal income (C$30,000–39,999 vs. census population mean = C$30,504.16, SD = C$37,808.12). Since our sample was recruited shortly after childbirth, there were predictably fewer non-intact families than in the general population (5% vs. 16.8% lone-parent families; 4.3% vs. 10.3% stepfamilies). The ratio of Canadian-born to immigrants was somewhat higher in the iKFP sample (57.7% vs. 47.6%), likely due to the language requirement for participation. Also, more study mothers had earned a bachelor’s degree or higher in the iKFP sample (53.3% vs. 30.6%). The sample was ethnically and socio-demographically diverse (see **Table 1**).

**Table 1:** Demographic characteristics of the sample at study entry (*N* = 501).

Measure	*N*	% of sample
Ethnicity of mothers European/Caucasian South Asian East Asian Black Other	501 283 73 60 46 39	100.0 56.5 14.6 12.0 9.2 7.7
Teenage mother	31	6.2
Single parent family	32	6.4
Immigrant family (mother not Canadian-born)	233	46.5
Low income family (<$20,000)	45	9.5
Mother’s years of education (<high school)	34	6.2
Mothers scoring in depressed range on CESD	71	14.4

*Total sample at wave 1, N = 501.*

At Time 1 (T1; *M*_age_ = 2.0 months; SD = 1.06), 501 families were enlisted in the study. Due to sample attrition, 397 (79.2%) families were followed up at Time 2 (T2; *M*_age_ = 1.60 years; SD = 0.16). Attrition analysis showed that dropout, similar to other longitudinal studies, was related to higher levels of social risk: maternal depression at T1, χ^2^ (*df* = 1) = 7.2, *p* = 0.01, being in a non-intact family, χ^2^ (*df* = 1) = 11.1, *p* = 0.002, immigrant status, χ^2^ (*df* = 1) = 13.5, *p* < 0.001, teenage parenthood, χ^2^ (*df* = 1) = 6.7, *p* = 0.02, maternal education <high school, χ^2^ (*df* = 1) = 10.5, *p* = 0.002, and family income< $20,000, χ^2^ (*df* = 1) = 7.1, *p* = 0.01. Of the 397 children remaining at T2, no social-cognitive data were available for 24 children due to non-compliance, lack of visibility (e.g., child went off camera), parent intrusion (e.g., directing child), non-administration due to family constraints (e.g., time limitations) or tester administration error (e.g., not following the standardized protocol). This resulted in a final sample of 373 children providing data on social cognition.

### Procedure

The study design combined the strengths of epidemiological methodology (large and diverse sample, multiple siblings, home visits) with the strength of developmental methodology (tasks developed in the laboratory, detailed microsocial observational data). At each time point, two trained interviewers visited each family’s residence for approximately 2 h. Data collection included questionnaires, age-appropriate developmental tasks for target children at T2, and observational measures of mother–child interactions at T2.

### Measures

#### Cumulative Biomedical Risk

At T1, mothers reported on their own pregnancy complications and a variety of infant birth problems. A single item was used to assess the presence/absence (0 = absent; 1 = present) of each of the following: (1) pregnancy diabetes; (2) hypertension; (3) thyroid problems (4) loss of fetal movement; (5) injury to the abdomen; (6) infant need for intensive care after birth; (7) infant need for oxygen/ventilation; and (8) infant need to be transferred to a specialized hospital. Further, two additional continuous perinatal risk factors were dichotomized based on pre-defined cut-points. These were: (9) low birth weight (<2500 g); and (10) short gestation (<37 weeks). A count of these biomedical risks was computed. The distribution of problems in the sample was as follows: 0 problems (68.0%), 1 problem (25.0%), 2 problems (4.4%), 3 problems (1.2%), 4 problems (1.2%), 5 problems (0%), and 6 problems (0.2%). No individuals reported 7–10 problems. Further, as few individuals existed in the upper tail of the distribution, we combined 4–6 problems into a category of ‘4 or more’ problems (1.4% of the sample). Thus, this variable represented a count of the number of biomedical risks/complications on a scale from zero to ‘4 or more.’

#### Maternal Responsivity

Observational data were gathered at T2 on mother–child interactions across three 5-min tasks: (1) unstructured free play with no toys; (2) a structured cooperative building task (using Duplo blocks to build a design from a picture); and (3) reading from a wordless picture book. For all three tasks, three domains of responsivity were coded using the Parent–Child Interaction System of global ratings (PARCHISY, Deater-Deckard et al., unpublished) and the Coding of Attachment Related Parenting (CARP, Matias, 2006). *Sensitivity* (from the CARP) measured the degree to which the parent responded to the child’s verbal and non-verbal signals, supported the child’s autonomy, showed warmth, and demonstrated an ability to see things from the child’s perspective. *Mutuality* (from the CARP) is a dyadic code and is compatible with the concept of the ‘goal-corrected partnership’ (Bowlby, 1982). Mutuality was indexed by reciprocity in conversation (e.g., a conversation that “goes somewhere” and is a genuine dialog), affect sharing, joint engagement in task, and open body posture. Finally, *positive control* (from the PARCHISY) captures the parents’ positive means of getting the child to do something that she wanted him or her to do through the use of praise, explanations, and open ended questions. Each of these three domains – sensitivity, mutuality, and positive control – was rated on a 7-point scale for each of the three tasks. Internal consistency of the measures was high (α = 0.85). Thus, a composite measure of ‘maternal responsivity’ was created by averaging the sensitivity, mutuality, and positive control scores across all three tasks. Higher scores reflected higher levels of maternal responsivity. Coders were trained to criterion and then 10% of the interactions were double-coded. Reliability was checked throughout the coding period to guard against rater drift. Inter-rater reliability was high (α = 0.94). Coders were blind to the biomedical history of the children.

#### Social Cognition

This was measured at T2 (18 months) by four independent observational tasks assessing children’s joint attention, empathy, cooperation, and self-recognition. Each of these tasks was previously validated and widely used in laboratory studies, and we adapted these for use in the home interviews. A complete description of these tasks can be found in Supplementary Material, as well as Wade et al. (2014c). Briefly, in the joint attention task children were required to respond to an adult interviewer’s bids for directing their attention (Mundy et al., 2003); in the empathy task (Kochanska et al., 1994) children were assessed for their ability to respond to the feigned distress of the interviewer; in the cooperation tasks (Warneken et al., 2006) children had to work collaboratively with the interviewer toward a shared goal; and in the self-recognition task we evaluated children’s ability to recognize the objectivity of their body using the mirror-rouge paradigm (Amsterdam, 1972). Inter-rater reliabilities across tasks were good: α = 0.94 for joint attention, α = 0.82 for empathy, α = 0.86 for cooperation, and κ = 0.79 for self-recognition. Scores on these measures were submitted to a confirmatory factor analysis (CFA), consistent with their ostensible coherence as indicators of children’s latent social cognition (Wade et al., 2014c). Model fit for the *social cognition* factor was excellent in accordance with Hu and Bentler’s (1999) recommended cut-offs: root-mean-square-error of approximation (RMSEA) = 0.023, comparative fit index (CFI) = 0.99, and standardized root-mean-square residual (SRMR) = 0.021. Model-estimated loadings were also positive and significant at the *p* < 0.001 level for all indicators. Factor scores were saved and used as the primary outcome variable. The *social cognition* factor was normally distributed with a mean of zero.

#### Covariates

Based on previous studies demonstrating the association between certain socio-demographic and constitutional factors and social cognition, a number of variables were controlled for: (1) child age in years; (2) child gender (0 = male; 1 = female); (3) annual family income, assessed on a scale from 1 (‘no income’) to 16 (‘$105,000 or more’); (4) maternal education, assessed as the total number of years of formal schooling, not including kindergarten; (5) immigrant status of the mother (i.e., 0 = immigrant; 1 = born in Canada); (6) maternal depression, assessed using the Center for Epidemiological Studies Depression Scale (CES-D; Radloff, 1977), a widely used self-report scale that assesses depression in non-clinical populations; and (7) children’s language ability, measured concurrent with social cognition (18 months) using the MacArthur-Bates Communicative Development Inventories (CDIs; Fenson et al., 1994).

### Statistical Analysis

First, all predictor and covariate variables were standardized, and the interaction term between cumulative biomedical risk and maternal responsivity was computed by multiplying the *z*-scores of these two variables (Preacher and Rucker, 2003). We then performed hierarchical multiple regression using MPlus 7.0. To handle variable amounts of missing data, we used full-information maximum likelihood estimation (FIML), which produces unbiased parameter estimates and SEs when data are missing at random (Enders and Bandalos, 2001). The model was fitted using the maximum likelihood with robust SEs estimator (MLR), which gives parameter estimates with SEs and a chi-square that are robust to non-normality (Yuan and Bentler, 2000). In the first step of the multiple regression analysis, the covariates were entered into the model. In the second step, the covariates plus the main effects of cumulative biomedical risk and maternal responsivity were entered into the model. Finally, in the third step, the interaction between biomedical risk and maternal responsivity was added to the variables from all previous steps in order to determine whether the interaction term predicted social cognition above and beyond covariates and main effects.

## Results

### Preliminary Descriptive Analysis

**Table 2** presents the descriptive statistics for all study variables, including bivariate associations. Notable associations in **Table 2** include the positive relationship between social cognition and child age, female gender, family income, language ability, and maternal responsivity, as well as the negative association between social cognition and cumulative biomedical risk. Higher biomedical risk was also associated with lower socioeconomic status (family income and maternal education), as well as higher levels of maternal depression and lower levels of maternal responsivity. Maternal responsivity was associated with nearly all other study variables. A preliminary trend analysis showed that there was a significant linear association between cumulative biomedical risk and social cognition, B (SE) = -0.02 (0.01), *p* = 0.047. Neither the quadratic, B (SE) = 0.01 (0.01), *p* = 0.10, nor the cubic trend, B (SE) = -0.01 (0.01), *p* = 0.22, were significant, suggesting that as cumulative biomedical risk increases, social cognition decreases in a linear fashion (see Supplementary Figure S1 for a plot of this association). Also, Supplementary Table S1 outlines the inter-relations between individual risk variables in the cumulative risk index. This Table shows a combination of independent and inter-dependent risk variables, making the cumulative risk approach suitable (see Evans et al., 2013).

**Table 2:** Descriptive statistics and correlations between study variables.

	1	2	3	4	5	6	7	8	9	*M* or %	SD
(1) Child age	-									1.60	0.16
(2) Female gender	0.00	-								49.3	-
(3) Family income	0.05	0.02	-							11.9	4.06
(4) Maternal education	0.11^∗^	-0.04	0.51^∗∗∗^	-						15.3	2.68
(5) Immigrant status	-0.14^∗∗^	-0.02	0.34^∗∗∗^	0.13^∗∗^	-					46.5	-
(6) Maternal depression	-0.06	-0.02	-0.32^∗∗∗^	-0.25^∗∗∗^	-0.17^∗∗∗^	-				9.46	7.29
(7) Language ability	0.00	0.00	0.04	0.02	0.12^∗^	0.04	-			0.00*^a^*	1.00
(8) Maternal responsivity	0.07	0.14^∗∗^	0.34^∗∗∗^	0.26^∗∗∗^	0.24^∗∗∗^	-0.16^∗∗^	0.17^∗∗^	-		3.51	0.79
(9) Social cognition factor	0.42^∗∗∗^	0.14^∗∗^	0.13^∗^	0.06	0.02	-0.05	0.27^∗∗∗^	0.24^∗∗∗^	-	0.00*^a^*	0.14
(10) Biomedical risk	-0.01	-0.02	-0.18^∗∗∗^	-0.17^∗∗∗^	0.04	0.10^∗^	-0.02	-0.12^∗^	-0.10^∗^	-*^b^*	-

*^∗∗∗^p < 0.001, ^∗∗^p < 0.01, ^∗^p< 0.05, p < 0.10.*

*^a^These are either standardized scores or factor scores with a mean of zero.*

*^b^See in-text for the distribution of this variable.*

### Primary Regression Analysis

We performed hierarchical multiple linear regression to examine the effect of cumulative biomedical risk, maternal responsivity, and their interaction on social cognition. These results are presented in **Table 3** In the first step of the model, covariates that were shown to be significant predictors of social cognition at 18 months included age, female gender, and child language ability. Family income was marginally associated with social cognition. None of the other covariates were significant predictors. This step of the model accounted for a significant 30% of the variance in social cognition. In the second step of the model, above and beyond covariates, there was, there was a significant main effect of cumulative biomedical risk and a marginally significant main effect of maternal responsivity on social cognition. This model accounted for an additional 2.1% of the variance in social cognition, or 32.1% overall. Finally, in the third step of the model, over and above covariates and main effects, the interaction between cumulative biomedical risk and maternal responsivity significantly predicted social cognition. The main effects of both biomedical risk and maternal responsivity were reduced to non-significance upon inclusion of the interaction term. This model accounted for a total of 32.8% of the variance in social cognition.

**Table 3:** Model results for the primary multiple regression analysis.

	β	SE	*p*	*R^2^*	*F* change
**Step 1:**
Female gender	0.30	0.09	0.001	0.30^∗∗∗^	20.49^∗∗∗^
Child age	0.44	0.05	<0.001			
Family income	0.09	0.05	0.10			
Maternal education	-0.02	0.06	0.77			
Child language ability	0.27	0.05	<0.001			
Canadian-born	0.10	0.10	0.33			
Maternal depression	-0.01	0.05	0.76			
**Step 2:**
Cumulative biomedical risk	-0.12	0.06	0.043	0.32^∗∗∗^	3.95^∗^
Maternal responsivity	0.10	0.05	0.051		
**Step 3:**
Cumulative biomedical risk^∗^maternal responsivity	0.10	0.05	0.047	0.33^∗∗∗^	3.29^†^

*^†^p < 0.10, ^∗^p < 0.05, ^∗∗^p < 0.01, ^∗∗∗^p < 0.001.*

*R^2^ – Cumulative R^2^.*

### Follow-Up Analysis of Simple Slopes

**FIGURE 1:**
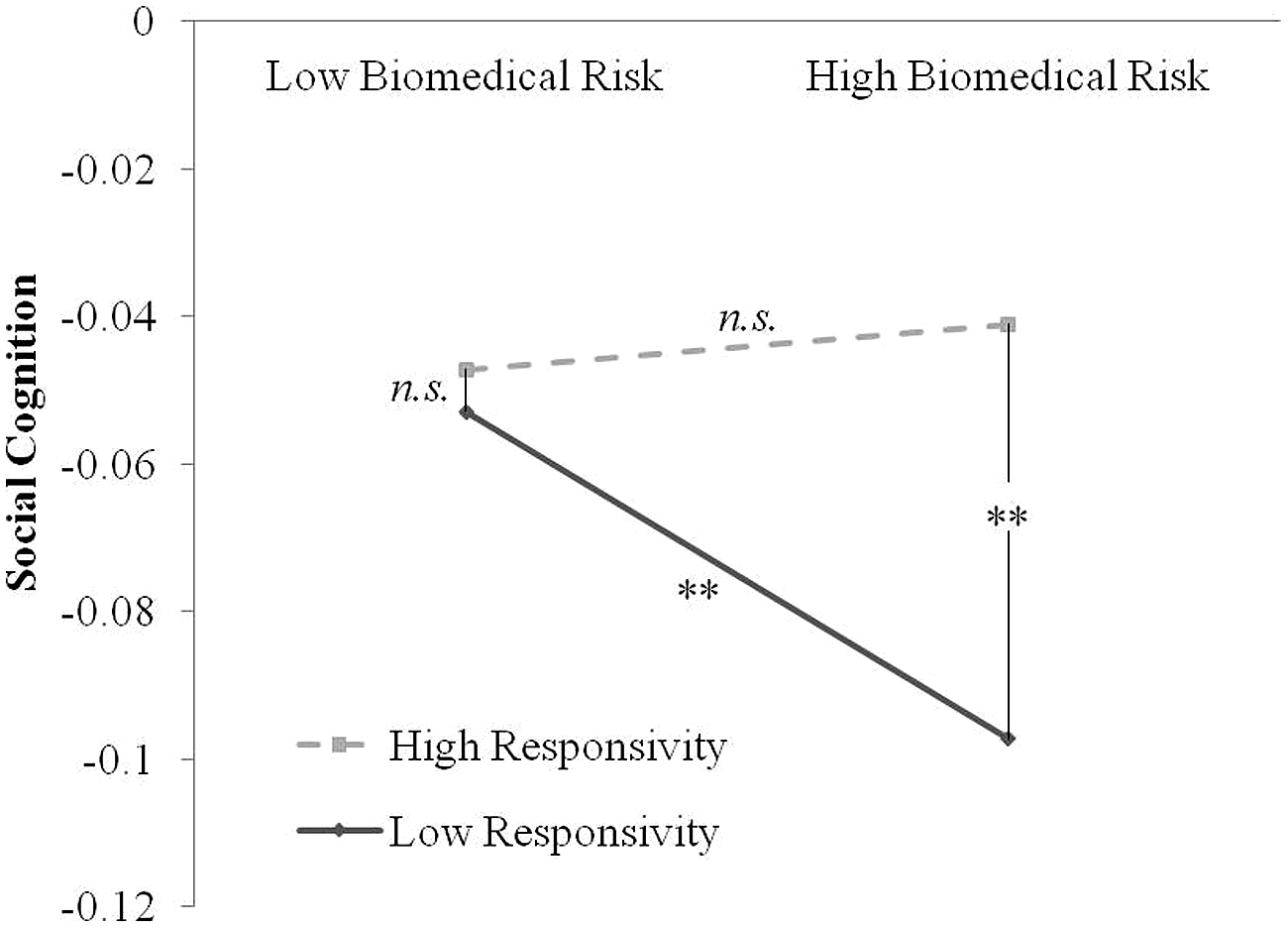
**Plotted interaction between cumulative biomedical risk by responsive parenting on social cognition at 18 months**. Solid line represents low levels of maternal responsivity (-1 SD below the mean), and hashed line represents high levels of maternal responsivity (+1 SD above the mean). Each point on the plot represents a combination of high/low biomedical risk and high/low responsivity, for a total of four possible combinations. ^∗∗^denotes that that comparison between points is significant, where *n.s.* denotes that there is no difference between the points on social cognition.

To explicate the pattern of the interaction between biomedical risk and maternal responsivity, we performed an analysis of simple slopes, which tests the relationship between biomedical risk and social cognition at different levels of the moderator (Aiken and West, 1991). In the case of a continuous moderator (i.e., responsive parenting), the common approach to examine the regression relationship at high (+1 SD) and low (-1 SD) levels of the moderator (Cohen et al., 2013). The pattern of this interaction can be seen in **Figure 1**. This figure shows that, when biomedical risk is low, there was a minimal effect of responsivity on social cognition (*z* = 0.38, *p* = 0.71). Alternatively, at high levels of biomedical risk, responsivity was positively related to social cognition (*z* = 2.66, *p* = 0.008). Examining the converse associations, at low levels of responsivity, biomedical risk was strongly negatively associated with social cognition (*z* = -2.70, *p* = 0.002), while at high levels of responsivity, biomedical risk was not associated with social cognition (*z* = 0.38, *p* = 0.70).

## Discussion

The aim of the current study was to investigate the association between cumulative biomedical risk and social cognition at 18 months, and whether maternal responsivity moderated this association. It was shown that, above and beyond covariates, both maternal responsivity and cumulative biomedical risk independently predicted social cognition at 18 months. Further, consistent with study hypotheses, maternal responsivity was shown to moderate the association between biomedical risk and social cognition, with the effect of biomedical risk only apparent at low levels of maternal responsivity. Alternatively, at high levels of maternal responsivity, there was no effect of cumulative biomedical risk on social cognition. These results provide the first empirical evidence that accumulating biomedical risk factors may be one source of inter-individual variability in children’s social-cognitive skills in the second year of life. Also, and consistent with risk-resiliency models of development, these findings suggest that postnatal socialization factors – specifically responsive caregiving – may protect against the impact of early biomedical risk on child outcomes.

Our finding that responsive parenting acts as a protective factor against early biomedical complications is consistent with intervention studies showing that cognitive and social outcomes of perinatally at-risk children may be fostered through training programs that build parents’ cognitive and affective responsiveness (Landry et al., 1997, 2006, 2008, 2012). In general, these studies show that intervention effects on broad cognitive and socio-emotional competence operate through changes in parenting behaviors, and these effects are strongest in the most biologically at-risk children (e.g., very low birth weight, preterm). Within the context of these intervention studies, the current findings are noteworthy for two reasons: first, they show that, in addition to individual biological insults such as low birth weight, the accumulation of early biomedical risk factors may also compromise children’s emerging social-cognitive skill development, operationalized within a framework that posits underlying capacities for self-other differentiation and understanding of intentions (see also Moore, 2007; Wade et al., 2014c); second, they demonstrate that the protective role of responsive maternal behaviors is also present within a normative, epidemiological sample of children with varying degrees of biological risk. Within such a sample, the presence of individual biomedical risks are typically not powerful individual predictors of child outcomes, either because these are low frequency events, or because there are a host of identified or unidentified factors that buffer the effect of isolated risks. Rather, it may be that the accumulation of multiple biomedical risks is what creates meaningful differences in children’s social cognition within the general population.

The mechanisms through which biomedical risks influence children’s social cognition are presumed to involve changes in infant brain development. However, little research exists to support the idea that prenatal/birth insults specifically impact the neural regions that support social cognition in humans. The postnatal progression following such biomedical risks may shed light on the mechanisms that underlie differences reported here. Infants born with prenatal/perinatal complications are at a higher risk for postnatal complications (e.g., metabolic complications; Lubchenco and Bard, 1971; Hendderson et al., 2006). Experimental evidence from animal models demonstrates that all these factors can stimulate or precipitate neuronal death in the infant brain resulting in volume loss in particular regions within the brain (Bhutta and Anand, 2001). This is supported by findings from Peterson et al. (2000), who examined brain volume differences in 8-year-old children born with birth complications. This study demonstrated smaller volumes in the amygdala, hippocampus, basal ganglia, and cortical regions, all of which were associated with increased risk of ADHD and lower cognitive scores. Some of these regions have also been implicated in social cognition (Adolphs, 2001). Further, in a notable study by Carmody et al. (2006), cumulative medical and environmental risk was shown to be associated with lower cognitive performance in adolescence, as well distinct patterns as brain activation in temporal and parietal cortical regions. This is interesting given that social cognition, including the capacity for self-other differentiation and mental-state inference, is believed to be supported by a distributed neural network that includes temporal and parietal areas (Decety and Sommerville, 2003; Van Overwalle, 2009). By extension, it is plausible that accumulating biomedical risks are associated with social cognition by virtue of their effect on functional brain networks during *in utero* and early postnatal development. Moreover, recent studies suggest the possibility that the strongest associations between pre/perinatal characteristics and brain development may exist within the normal range (Raznahan et al., 2012; Walhovd et al., 2013). The current results show that, indeed, meaningful differences in social cognition may exist as a function of normal variation in summative biomedical complications. Despite these interesting findings, the exact mechanism(s) connecting biomedical risk, neural development, and social cognition require future research.

Perhaps most interesting to the current study was the finding that responsive parenting moderated the association between cumulative biomedical risk and social cognition. These results are consistent with other observational studies on the protective effect of positive caregiving on children’s varied behavioral and mental health outcomes (Raine et al., 1994, 1997; Landry et al., 1997; Laucht et al., 1997, 2001; Voigt et al., 2013). Schore’s *regulation theory* suggests that positive parent–child interactions help promote adaptive functioning through regulation of neurobiological processes, including structural and functional neuroanatomy (Schore, 1996, 2001). Moreover, regulation theory posits a maturational process from prenatal to postnatal development, consistent with the notion that there is substantial brain development over the first 2 years of life (Knickmeyer et al., 2008). The developing brain is also very vulnerable to both environmental insult and enrichment, the latter of which may promote some the protective effects of responsive caregiving. Interestingly, recent findings from longitudinal studies show that the provision of early responsive caregiving is associated with enhanced physiological organization and resultant cognitive functioning over the first 10 years of life (Feldman et al., 2014). The precise role of responsive parenting, including the specific forms of care that foster neurobiological development and social cognition, requires further investigation. However, collaborative evidence from the fields of pediatrics, developmental psychology, and social neuroscience point to the importance of early responsive care in ameliorating the long-term sequelae of adverse pre/perinatal events on neurological and cognitive morbidity. Indeed, small variations in biological risk may create momentous gaps in children’s social and cognitive development, and these effects may persist across the lifespan in the absence of interventions that target foundational inter-personal transactions with caregivers early in postnatal life (Walhovd et al., 2014).

The results of this study should be considered in light of several strengths and limitations. The strengths included the prospective, multi-method, longitudinal design, large and diverse sample, and use of detailed observational outcome data on 18-month social-cognitive measures. Inclusion of numerous socio-demographic confounding variables also adds to the robustness of the current findings. In regard to limitations, the current Canadian sample was more advantaged than the general population, and participation was restricted to children born >1500 g. These sampling factors may limit the generalizability of the results. Also, each of the 10 biomedical risks was low frequency, measured through maternal report, and typically dichotomous. Agreement between self-report and criterion-standard medical record data has been shown to be high for prenatal complications (Okura et al., 2004) and other pre/perinatal events (Lederman and Paxton, 1998; Tomeo et al., 1999). However, future studies using more comprehensive information from obstetrical records would strengthen these findings. Moreover, additional information on the timing and severity of particular prenatal conditions (e.g., diabetes, hypertension, thyroid problems), as well as the specific reasons neonatal specialized care was needed (e.g., ischemia, anoxia, hematological problems), would improve suggestions about the mechanisms at play. More extensive records of prenatal care – which were not available in the current epidemiological study – would also shed light on the nature of these influences on child outcomes. Also, although significant, the effects documented herein were generally small in magnitude, suggesting that there are additional sources of unexplained variability in social cognition worthy of future investigation. Likewise, biomedical risk and responsive parenting were not completely independent predictors of social cognition, leading to the possibility that heightened biomedical risk may also predict variability in parenting. Possible mechanisms that link early biomedical risk to both parenting and child behavior – for instance, through the use of longitudinal cross-lagged mediation models – may be useful in elucidating these pathways to social cognition. On a related note, the fact that social cognition and maternal responsivity were measured contemporaneously (i.e., both at 18 months) precludes inferences about causality, and additional studies are warranted to determine the directionality of effects. Finally, although cumulative risk indices are powerful measures for examining the extent of risk exposure on developmental outcomes, future studies comparing the utility of these metrics to individual risk factors (measured through client records or direct measurement of risk, e.g., degree of hypoxia, level of hyperglycemia or hypertension, length of time in specialized care, etc.), are warranted based on these preliminary results.

## Conflict of Interest Statement

The authors declare that the research was conducted in the absence of any commercial or financial relationships that could be construed as a potential conflict of interest.
