# Supplementary material for: Cumulative biomedical risk and social cognition in the second year of life: prediction and moderation by responsive parenting
Source: Front Psychol. 2015 Apr 1;6:354. doi: 10.3389/fpsyg.2015.00354 (PMC4381485; doi:10.3389/fpsyg.2015.00354)
Supplement: Supplementary file 1 [file table_1.pdf]

Supplementary Material

Cumulative biomedical risk and social cognition in the second year of life: prediction and moderation by responsive parenting

Mark Wade[1]*, Sheri Madigan[1], Jennifer M. Jenkins[3]

[1]Department of Applied Psychology and Human Development, University of Toronto, Toronto, Ontario, Canada.

*** Correspondence:** Mark Wade, Department of Applied Psychology and Human Development, University of Toronto, 252 Bloor Street West, Toronto, Ontario, M5S 1V6, Canada.
wadem2@gmail.com

1. Supplementary Figures and Tables

1.1. Supplementary Tables

Supplementary Table 1. Additional measurement information on social cognition.

For the *Social Cognition* factor, four independent observational tasks were used to assess children's joint attention, empathy, cooperation, and self-recognition at T2 (18 months). These tasks were administered during the home visit. Each task is described below:

**Social Cognition Factor**	
**Measure**	**Description**
Joint Attention	Assessed as children's ability to follow the gaze of an adult interviewer (Carpenter, Nagell, & Tomasello, 1998), using a task from the Early Social Communication Scale (ESCS; Mundy, et al., 2003). The child sat with thier mother across from the experimenter. Two colorful pictures were placed beside the child, and two behind. The tester first ensured she had the attention of the child by calling the child's name, tapping the table, or gently touching the child. She then pointed to the four posters in a systematic order: tester's left, left-behind, right, and right-behind. The point consisted of the tester turning her entire torso, visually orienting to the poster. During the pointing trial, if the child did not immediately redirect their attention to the poster, the tester proceeded to say the child's name three times. If the child still did not redirect his/her attention, the tester paused before redirecting attention to the child. This task was administered twice, separated by another activity, for a total of 8 possible 'respond to joint attention' (RJA) observations for each child. A trained coder viewed videotapes and coded children's ability to redirect attention to the posters on a 4-point scale. If the child immediately redirected attention to the poster after the tester's point, the child received a score of 4. If the child redirected attention after their name was said by the tester, they received a score of 3. If the child delayed redirection of attention until after the tester's point was finished, but before the next trial commenced, they received a score of 2. If the child failed to redirect attention, they received a score of 1. Inter-rater reliability was high (α = .94). A task analysis

	revealed significant mean differences between side point (*M* = 3.90, *SD* = .27) and behind point (*M* = 3.30, *SD* = .91) trials ($t(279) = 11.8$, $p < .01$), suggesting side points were easy for most 18-month-olds. Furthermore, only behind points correlated significantly with children's concurrent vocabulary ($r = .22$, $p < .01$), indicating better construct validity for the behind-point trials. Thus, for each child, only the four observations of the behind trials were used as the measure of RJA. We took the mean score for each of the four trials, resulting in a maximum mean score of 4.
Empathy	This was measured as the child's responsiveness to the feigned distress of an adult confederate. At regular points throughout the home visit, the interviewer pretended to hurt her knee and finger, and to drop and ostensibly break her favourite toy ('Mickey', a toy monkey). Two coders watched all empathy events and rated children on six statements based on their reactions. A thin-slice coding method was used (Ambady, Bernieri, & Richeson, 2000; Ambady & Rosenthal, 1992). This is a global or impressionistic rating of the child's behaviour, and has been used successfully in rating child behaviour (Prime, Perlman, Tackett, & Jenkins, 2011). Based on this methodology, coders are encouraged to make general judgements using all information available from the empathy events, and their final ratings are averaged to decrease the impact of a single observer's judgment (Ambady, et al., 2000). Ratings were based on an adaptation of an empathy scale developed by Kochanska (Kochanska, DeVet, Goldman, Murray, & Putnam, 1994). Five items were removed from that scale because they were not relevant to the current tasks (e.g. reactions to movie characters or animals being hurt). Items included: 'Will try to comfort or reassure another in distress'; 'Likely to offer toys or candy to crying playmate even without parental suggestion'; 'Can tell at just one glance how others are feeling'; 'Likely to ask what's wrong when seeing someone in distress'; 'Will feel sorry for other people who are hurt sick or unhappy'; and 'Is not likely to become upset if a playmate cries'. These were each rated on a 7-point scale from 1 ('extremely untrue') to 7 ('Extremely true'). The internal consistency was .98 for coder 1 and .96 for coder 2. Although inter-rater agreement using thin-slice methodology is not usually reported, agreement between coders was high ($\alpha = .82$).

Cooperation	Cooperation skills were measured with two previously-developed tasks: trampoline and double tubes (Warneken, Chen, & Tomasello, 2006). These assessed the degree to which children cooperate with an adult examiner towards a shared goal. The tasks require the child to change his/her behaviour to succeed. Four measures were taken: (1) Trampoline task: the child was invited by the tester to help make a bear dance on a hand-held trampoline. If the child failed to cooperate by not holding up their end, the trampoline would collapse. The first 10 seconds were allowed as a learning phase, and were not coded. Subsequently, 10-second intervals were coded on a 5-point scale, to a maximum of 80 seconds (8 total intervals). The scale ranged from 1 (‘no success’) to 5 (‘high engagement’). A mean of the intervals was taken. (2) Double tubes task: the child was invited to assist the experimenter complete a sequence of actions in which a ball was rolled down one of two tubes, and the child was required to catch the ball at the bottom. In contrast to the trampoline task, the child needed to perform a different but complementary action to the tester in order to achieve the goal. That is, to be successful, the child could not simply imitate the tester. The first catch trial was allowed as a learning phase, and was not coded. Subsequently, each catch invitation was coded on a 5-point scale, with a maximum of 8 trials coded. The scale ranged from 1 (‘no attempt’) to 5 (‘complete success’). A mean of the 8 trials was computed for each child. (3) After the trampoline and double tubes tasks, coders rated a global cooperation score for each task. This score was coded on a 4-point scale, based on the percentage of time the child cooperated (0-25%, 26-50%, 51-75%, 76-100%). A mean across the two tasks was computed. Finally, coders rated the number of times the child was uncooperative throughout the (4) trampoline and (5) double tubes tasks. Ratings were on a scale from 0 (‘none’) to 3 (‘3 or more times’). These items were reverse coded. Ten percent of videotapes were double coded by independent coders. The mean inter-rater reliability across all cooperation tasks was $\alpha = .86$ (ranging from .68 to .96). A confirmatory factor analysis revealed that items loaded significantly onto the same factor, which explained 47% of the variance, with item loadings ranging from .54 to .76. A composite cooperation variable was constructed by taking the mean of the standardized scores across all cooperation measures. Individual measures were as follows: (1) Trampoline (M = 2.39, SD = 1.07); (2) Double Tubes (M = 3.41, SD = 1.06); (3) Global rating (M = 1.47, SD = .73); (4) Uncooperative trampoline (M = 1.86, SD = .99); Uncooperative double tubes (M = 1.89, SD = 1.06). All bivariate correlations were significant at $p < .01$. Internal consistency of the items in the composite was $\alpha = .71$.
Self-Recognition	During the cooperation task, interviewers covertly marked the child with a large coloured sticker at the front of their head on the hair. Children could not feel the sticker being placed. The child was then placed in front of a mirror and allowed to look at themselves for 30 seconds. If the child reached for the sticker or verbally acknowledged its presence (with or without prompting), this was take as evidence for self-recognition and the child received a score of 1. If the child did not recognize the sticker at all, they received a score of 0. Thus, this was a conservative estimate of children’s ability to recognize themselves in the mirror. Reliability on this task was $\kappa = 0.79$.

Supplementary Table 2: Relationship between individual risk variables in the cumulative risk index

	1.	2.	3.	4.	5.	6.	7.	8.	9.
1. Hypertension	-								
2. Diabetes	1.54	-							
3. Thyroid	.10	1.06	-						
4. Loss fetal movement	.20	.09	.82	-					
5. Injury to abdomen	1.52	.45	2.82	.93	-				
6. Intensive care	.003	5.55*	.10	.79	.34	-			
7. Ventilation/ oxygen	.01	.59	.69	.24	.30	145.7***	-		
8. Specialized hospital	.10	6.89	.07	2.89	.03	41.6***	48.2***	-	
9. Low birth weight	.91	.01	.62	.01	.27	49.6***	41.4***	.08	-
10. Short gestation	.01	.59	.69	.89	.30	77.5***	52.1***	11.1†	78.8***

***$p < .001$. **$p < .01$. *$p < .05$. †$p < .10$.

Note. These are Pearson chi-square statistics, as the variables were all dichotomous (yes/no).

1.2. Supplementary Figures

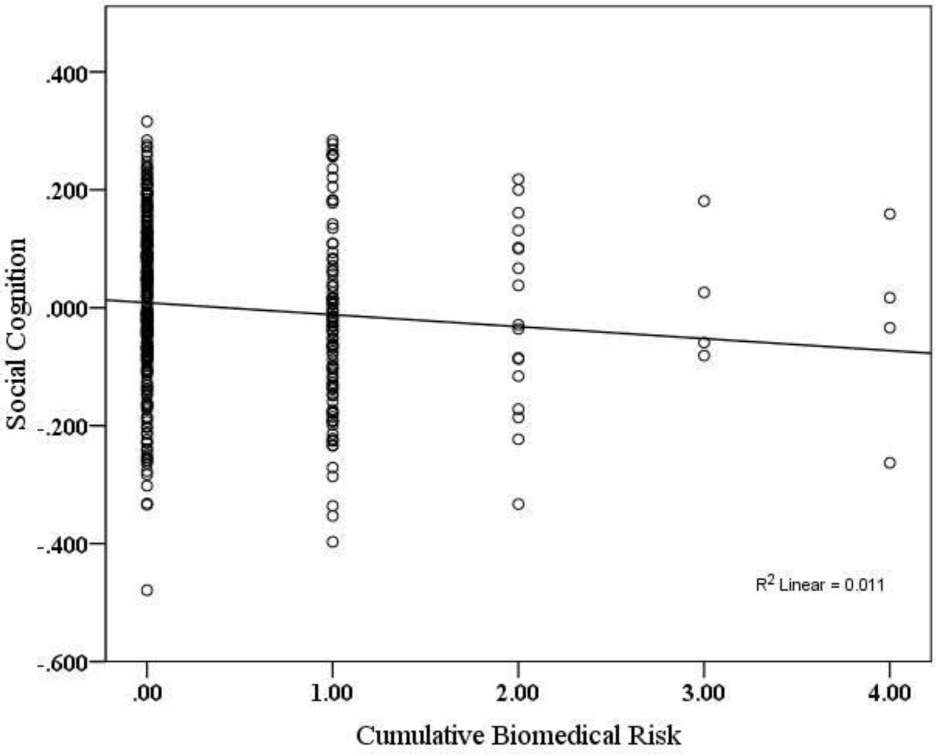

Supplementary Figure 1. Scatter plot of the association between cumulative biomedical risk and social cognition at 18 months. See in-text for the statistical trend analysis documenting the linear association.

2. References[1]

Ambady, N., Bernieri, F. J., & Richeson, J. A. (2000). Toward a histology of social behavior: Judgmental accuracy from thin slices of the behavioral stream. *Advances in experimental social psychology*, *32*, 201-271.

Ambady, N., & Rosenthal, R. (1992). Thin slices of expressive behavior as predictors of interpersonal consequences: A meta-analysis. *Psychological bulletin, 111*(2), 256.

Carpenter, M., Nagell, K., & Tomasello, M. (1998). Social cognition, joint attention, and communicative competence from 9 to 15 months of age. *Monographs of the Society for Research in Child Development., 63 (4).*

Kochanska, G., DeVet, K., Goldman, M., Murray, K., & Putnam, S. P. (1994). Maternal reports of conscience development and temperament in young children. *Child Development*, *65*(3), 852-868.

Mundy, P., Delgado, C., Block, J., Venezia, M., Hogan, A., & Seibert, J. (2003). Early Social Communikation Scales (ESCS). *Coral Gables, FL: University of Miami*.

[1] Provide the doi when available, and ALL complete author names.

Prime, H., Perlman, M., Tackett, J., & Jenkins, J. M. (2011). The development and validation of a thin-slice approach for coding scaffolding between siblings. *In preparation*.

Warneken, F., Chen, F., & Tomasello, M. (2006). Cooperative activities in young children and chimpanzees. *Child Development*, *77*(3), 640-663.
